# LY6E Restricts Entry of Human Coronaviruses, Including Currently Pandemic SARS-CoV-2

**DOI:** 10.1128/JVI.00562-20

**Published:** 2020-08-31

**Authors:** Xuesen Zhao, Shuangli Zheng, Danying Chen, Mei Zheng, Xinglin Li, Guoli Li, Hanxin Lin, Jinhong Chang, Hui Zeng, Ju-Tao Guo

**Affiliations:** a Institute of Infectious Disease, Beijing Ditan Hospital, Capital Medical University, Beijing, China; b Beijing Key Laboratory of Emerging Infectious Disease, Beijing, China; c Baruch S. Blumberg Institute, Hepatitis B Foundation, Doylestown, Pennsylvania, USA; d Department of Pathology and Laboratory Medicine, Western University, London, Ontario, Canada; Hudson Institute of Medical Research

**Keywords:** LY6E, SARS-CoV-2, human coronavirus, viral entry

## Abstract

C3A is a subclone of the human hepatoblastoma HepG2 cell line with strong contact inhibition of growth. We fortuitously found that C3A was more susceptible to human coronavirus HCoV-OC43 infection than HepG2, which was attributed to the increased efficiency of virus entry into C3A cells. In an effort to search for the host cellular protein(s) mediating the differential susceptibility of the two cell lines to HCoV-OC43 infection, we found that ArfGAP with dual pleckstrin homology (PH) domains 2 (ADAP2), gamma-interferon-inducible lysosome/endosome-localized thiolreductase (GILT), and lymphocyte antigen 6 family member E (LY6E), the three cellular proteins identified to function in interference with virus entry, were expressed at significantly higher levels in HepG2 cells. Functional analyses revealed that ectopic expression of LY6E, but not GILT or ADAP2, in HEK 293 cells inhibited the entry of HCoV-O43. While overexpression of LY6E in C3A and A549 cells efficiently inhibited the infection of HCoV-OC43, knockdown of LY6E expression in HepG2 significantly increased its susceptibility to HCoV-OC43 infection. Moreover, we found that LY6E also efficiently restricted the entry mediated by the envelope spike proteins of other human coronaviruses, including the currently pandemic SARS-CoV-2. Interestingly, overexpression of serine protease TMPRSS2 or amphotericin treatment significantly neutralized the IFN-inducible transmembrane 3 (IFITM3) restriction of human coronavirus (CoV) entry, but did not compromise the effect of LY6E on the entry of human coronaviruses. The work reported herein thus demonstrates that LY6E is a critical antiviral immune effector that controls CoV infection and pathogenesis via a mechanism distinct from other factors that modulate CoV entry.

**IMPORTANCE** Virus entry into host cells is one of the key determinants of host range and cell tropism and is subjected to the control of host innate and adaptive immune responses. In the last decade, several interferon-inducible cellular proteins, including IFITMs, GILT, ADAP2, 25CH, and LY6E, had been identified to modulate the infectious entry of a variety of viruses. Particularly, LY6E was recently identified as a host factor that facilitates the entry of several human-pathogenic viruses, including human immunodeficiency virus, influenza A virus, and yellow fever virus. Identification of LY6E as a potent restriction factor of coronaviruses expands the biological function of LY6E and sheds new light on the immunopathogenesis of human coronavirus infection.

## Abstract (precis)

Virus entry into host cells is one of the key determinants of host range and cell tropism and is subjected to the control of host innate and adaptive immune responses. In the last decade, several interferon-inducible cellular proteins, including IFITMs, GILT, ADAP2, 25CH, and LY6E, had been identified to modulate the infectious entry of a variety of viruses. Particularly, LY6E was recently identified as a host factor that facilitates the entry of several human-pathogenic viruses, including human immunodeficiency virus, influenza A virus, and yellow fever virus. Identification of LY6E as a potent restriction factor of coronaviruses expands the biological function of LY6E and sheds new light on the immunopathogenesis of human coronavirus infection.

## INTRODUCTION

Coronaviruses (CoV) are a large family of enveloped positive-strand RNA viruses with broad host ranges and tissue tropism (1, 2). While four human CoVs, including HCoV-229E, HCoV-OC43, HCoV-NL63, and HCoV-HKU1, cause mild upper respiratory tract infections, three zoonotic CoVs have crossed species barriers since 2002 to infect humans and cause severe acute respiratory syndrome (SARS) (3, 4), Middle East respiratory syndrome (MERS) (5, 6), and coronaviral disease-19 (COVID-19) (7, 8), with the mortality rates of 10%, 30%, and 1% to 2%, respectively (9, 10). No vaccine or antiviral drug is currently available to prevent CoV infection or treat the infected individuals. The cross-species transmission of zoonotic CoVs presents a continuous threat to global human health (11, 12). Therefore, understanding the mechanism of CoV infection and pathogenesis is important for the development of vaccines and antiviral agents to control the current COVID-19 pandemic and prevent future zoonotic CoV threats.

CoV entry into host cells, a viral envelope spike-protein-driven process to deliver nucleocapsids across the plasma membrane barrier into the cytoplasm, is the key determinant of virus host range and plays a critical role in zoonotic CoV cross-species transmission (2, 13). The entry process begins by the binding of viral envelope spike proteins to their specific receptors on the plasma membrane, which triggers endocytosis to internalize the viruses into the endocytic vesicles. The cleavage of viral envelope spike proteins by endocytic proteases and/or endosomal acidification triggers the conformational change of spike proteins to induce the fusion of viral envelope with the endocytic membrane, and thus release nucleocapsids into the cytoplasm to initiate viral protein synthesis and RNA replication. While angiotensin-converting enzyme 2 (ACE2) is the bona fide receptor for SARS-CoV, SARS-CoV-2 and HCoV-NL63 (14–16), MERS-CoV and HCoV-229E use dipeptidyl peptidase-4 (DPP4) and CD13 (also known as aminopeptidase N) as their receptors, respectively (17, 18). However, HCoV-OC43 and HCoV-HKU1 bind to 9-O-acetylated sialic acids via a conserved receptor-binding site in spike protein domain A to initiate the infection of target cells (19). As the key determinant of cell tropism, host range, and pathogenesis, CoV entry is primarily controlled by interactions between the envelope spike glycoproteins and host cell receptors, as well as the susceptibility of spike glycoproteins to protease cleavage and/or acid-induced activation of membrane fusion (20, 21). For instance, SARS-CoV can use ACE2 orthologs of different animal species as receptors (22–26) and the efficiency of these ACE2 orthologs to mediate SARS-CoV cell entry is consistent with the susceptibility of these animals to SARS-CoV infection (27–30). In addition, the site and efficiency of CoV entry can also be differentially regulated by the expression of endosomal cathepsins, cell surface transmembrane proteases (TMPRSS), furin, and trypsin (31–35).

Interferons (IFNs) are the primary antiviral cytokines that mediate innate and adaptive immune control of virus infection by inducing hundreds of genes, many of which encode antiviral effectors (36). In the last decades, several IFN-inducible proteins, including three IFN-induced transmembrane (IFITM) proteins (37), gamma-interferon-inducible lysosome/endosome-localized thiolreductase (GILT) (38), 25-hydroxycholesterol hydrolase (25HC) (39), ArfGAP with dual pleckstrin homology (PH) domains 2 (ADAP2) (40), and lymphocyte antigen 6 family member E (LY6E) (41) have been identified to restrict or enhance the entry of a variety of viruses. Interestingly, while IFITM proteins inhibit the entry of all the other human CoVs, HCoV-OC43 hijacks human IFITM2 or IFITM3 as entry factors to facilitate its infection of host cells (42, 43). We also demonstrated recently that GILT suppresses the entry of SARS-CoV, but not other human CoVs (38). As reported herein, in our efforts to identify host factor(s) determining the differential susceptibility of two closely related human hepatoma cell lines to HCoV-OC43 infection, we found that LY6E potently suppresses the infectious entry of all the human CoVs, including the currently pandemic SARS-COV-2. Our study also revealed that LY6E inhibits CoV entry via a mechanism distinct from IFITMs.

## RESULTS

### C3A is more susceptible to HCoV-OC43 infection than its parental cell line HepG2.

**FIG 1:**
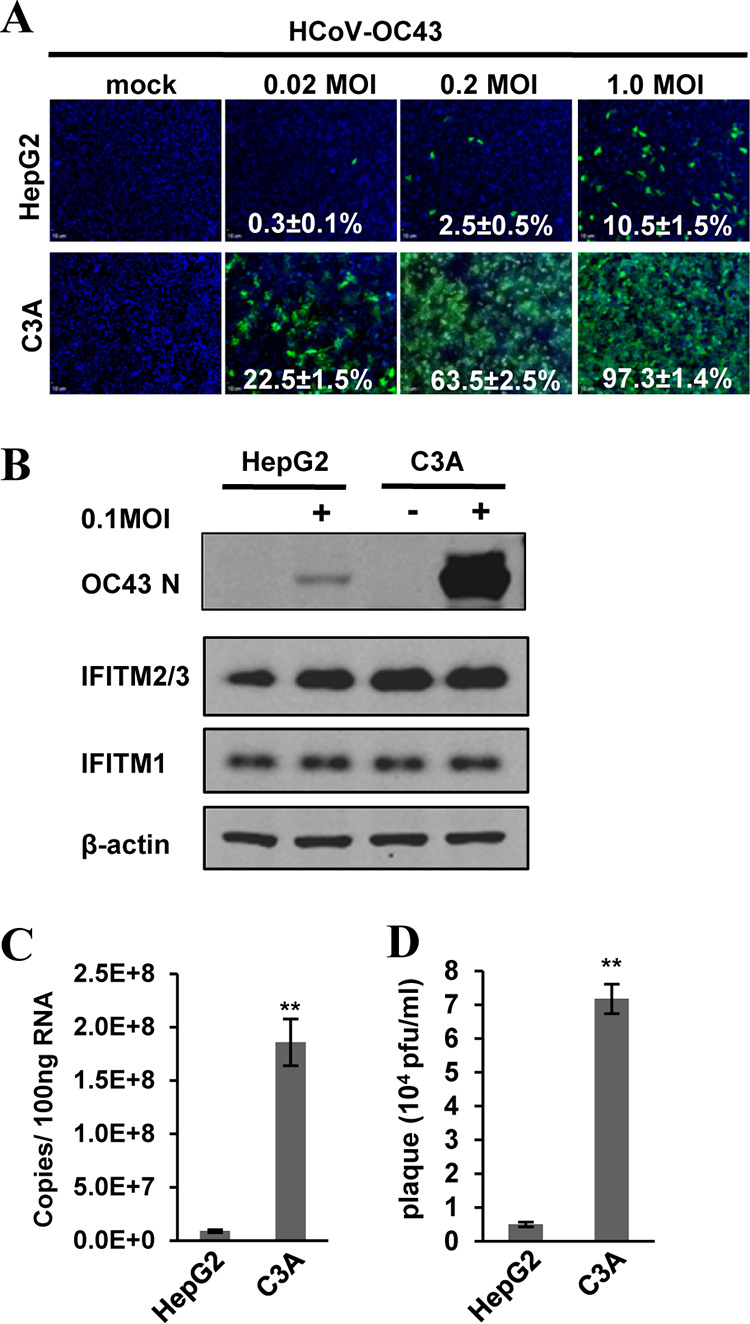
C3A cells are more susceptible to HCoV-OC43 infection than HepG2 cells. HepG2 and C3A cells were mock-infected or infected with HCoV-OC43 at the indicated MOI. (A) Cells were fixed at 24 h postinfection (hpi) and infected cells were visualized by indirect immunofluorescence (IF) staining of HCoV-OC43 N protein (green). Cell nuclei were visualized by DAPI staining. (B) HCoV-OC43 NP, IFITMs, and β-actin were determined by Western blotting assays. (C) Intracellular viral RNA was quantified by qRT-PCR assay and presented as copies per 100 ng total RNA. Error bars indicate standard deviations (*n* = 4). (D) Viral yields were determined with a plaque assay. Error bars indicate standard deviations (*n* = 4). **, *P* < 0.001 (Student's *t* test).

**FIG 2:**
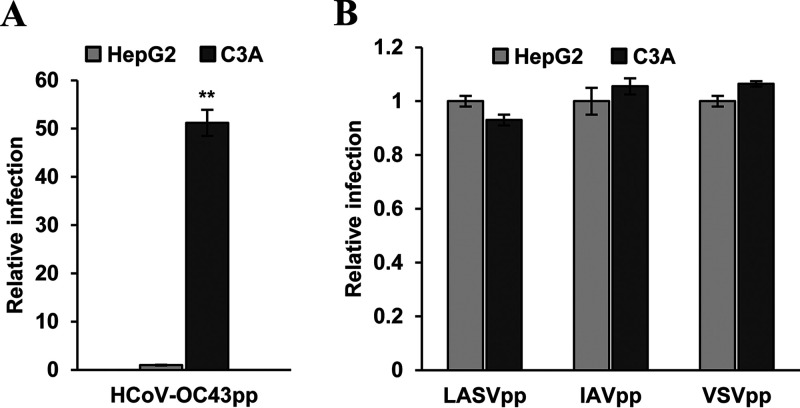
C3A cells support more efficient entry of lentiviral particles pseudotyped with HCoV-OC43 envelope proteins than HepG2 cells. HepG2 and C3A cells were infected with HCoV-OC43pp (A) or IAVpp, VSVpp, or LASVpp (B). Luciferase activities were determined at 72 hpi. Relative infection represents the luciferase activity from C3A normalized to that of HepG2 cells. Error bars indicate standard deviations (*n* = 6). **, *P* < 0.001 (Student's *t* test).

C3A is a subclone of HepG2 that was selected for strong contact inhibition of growth and high albumin production (44). Metabolically, C3A is more relevant to normal hepatocytes and has been used for development of bioartificial liver devices (45). Interestingly, we found that these two closely related cell lines drastically differ in their susceptibility to HCoV-OC43 infection (Fig. 1). Specifically, infection of the two cell lines with the virus at multiplicities of infection (MOI) of 0.02, 0.2, and 1 resulted in approximately 75, 25, and 10-fold more infected cells in C3A cultures than in HepG2 cultures at 24 h postinfection, respectively (Fig. 1A). Consistent with this finding, much higher levels of viral nucleocapsid protein (N) and RNA were detected in infected C3A cultures (Fig. 1B and C). Infected C3A cultures also produced approximately 20-fold more progeny viruses than HepG2 cultures did (Fig. 1D). To determine whether the differential susceptibility of the two hepatoma cell lines to HCoV-OC43 infection is due to a difference in virus entry or a postentry replication event, we compared the susceptibility of the two cell lines to lentiviral particles pseudotyped with envelope proteins of HCoV-OC43, influenza A virus (IAV), vesicular stomatitis virus (VSV), or Lassa fever virus (LASV) by using a luciferase assay to examine the entry efficacy. As shown in Fig. 2, while pseudoviral particles of IAV (IAVpp), VSV (VSVpp), and LASV (LASVpp) infected the two cell lines with similar efficiency, the efficiency of HCoV-OC43pp infection in C3A cultures is approximately 50-fold higher than that in HepG2 cultures. These results clearly indicate that the differential susceptibility is attributed to the distinct ability of the two cell lines to support the infectious entry of HCoV-OC43.

### IFITM proteins modulate HCoV-OC43 infection of C3A and HepG2 cells to a similar extent.

**FIG 3:**
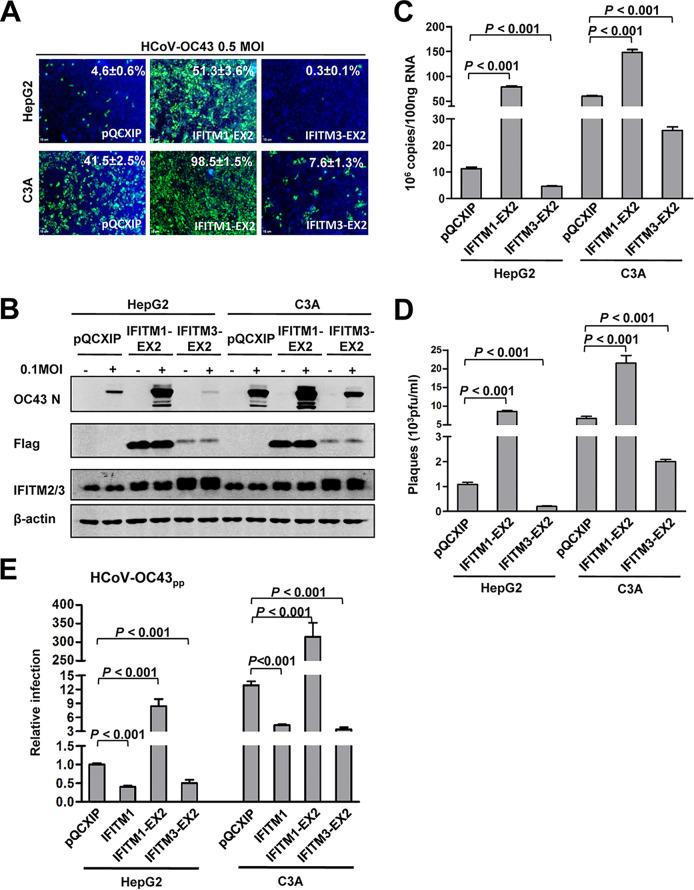
IFITMs modulate HCoV-OC43 infection of HepG2 and C3A cells to a similar extent and via the same mechanism. HepG2 and C3A cells were stably transduced with a control retroviral vector (pQCXIP) or a retroviral vector expressing an N-terminally FLAG-tagged IFITM1-EX2 or IFITM3-EX2. The resulting cell lines were infected with HCoV-OC43 at 0.5 MOI. (A) Cells were fixed at 24 hpi and virally infected cells were visualized by IF staining of HCoV-OC43 N protein (green). Cell nuclei were visualized by DAPI staining (blue). (B) HCoV-OC43 NP and exogenously expressed N-terminally FLAG-tagged IFITM proteins and total intracellular IFITM2/3 were determined by Western blotting assays with a monoclonal antibody against the FLAG tag and a rabbit polyclonal antibody against IFITM2/3. β-actin served as a loading control. (C) Intracellular viral RNA was quantified by a qRT-PCR assay and presented as copies per 100 ng total RNA. Error bars indicate standard deviations (*n* = 4). (D) Viral yields were determined with a plaque assay. Error bars indicate standard deviations (*n* = 4). (E) HepG2 and C3A stably transduced with a control retroviral vector (pQCXIP) or a retroviral vector expressing IFITM1, IFITM1-EX2, or IFITM3-EX2 were infected with HCoV-OC43pp. Luciferase activities were determined at 72 hpi. Relative infection represents the luciferase activity normalized to that of HepG2 cells transduced with empty vector (pQCXIP). Error bars indicate standard deviations (*n* = 6).

We reported previously that IFITM proteins differentially modulate HCoV-OC43 entry into target cells. While IFITM1 inhibits the virus entry, IFITM2 and IFITM3 enhance the cellular entry of this virus (42). To investigate whether the differential expression of IFITM proteins in the two cell lines is responsible for their difference in HCoV-OC43 infection efficiency, we examined IFITM protein expression by Western blotting assays and found the two hepatoma cell lines expressed similar levels of IFITM1 and IFITM2/3 (Fig. 1B). Because the C-terminal variable regions of IFITM1 and IFITM3 control the inhibition and enhancement of HCoV-OC43 entry (42), respectively, we further compared the effects on virus infection of overexpressing IFITM1-EX2, a mutant IFITM1 protein with its C-terminal domain replaced with the C-terminal domain of IFITM3 (42), and IFITM3-EX2, a mutant IFITM3 protein with its C-terminal domain replaced with the C-terminal domain of IFITM1 (42). As shown in Fig. 3, in spite of their distinct susceptibility, expression of IFITM1-EX2 and IFITM3-EX2 significantly enhanced and inhibited HCoV-OC43 infection of both cell lines, respectively, as evidenced by the significant changes in infected cell percentage (Fig. 3A), viral nucleocapsid protein expression (Fig. 3B), intracellular RNA accumulation (Fig. 3C), and yields of progeny virus (Fig. 3D). Moreover, pseudotyped lentiviral infection assays further demonstrated that IFITM1, IFITM1-EX2, and IFITM3-EX2 modulated HCoV-OC43 envelope-protein-mediated entry to a similar extent in the two cell lines (Fig. 3E). Accordingly, we concluded that IFITM proteins were not responsible for the observed differential susceptibility of the two hepatoma cell lines to HCoV-OC43 infection.

### LY6E inhibits the entry mediated by human CoV envelope spike proteins and is responsible for the differential susceptibility of C3A and HepG2 cells to HCoV-OC43 infection.

**FIG 4:**
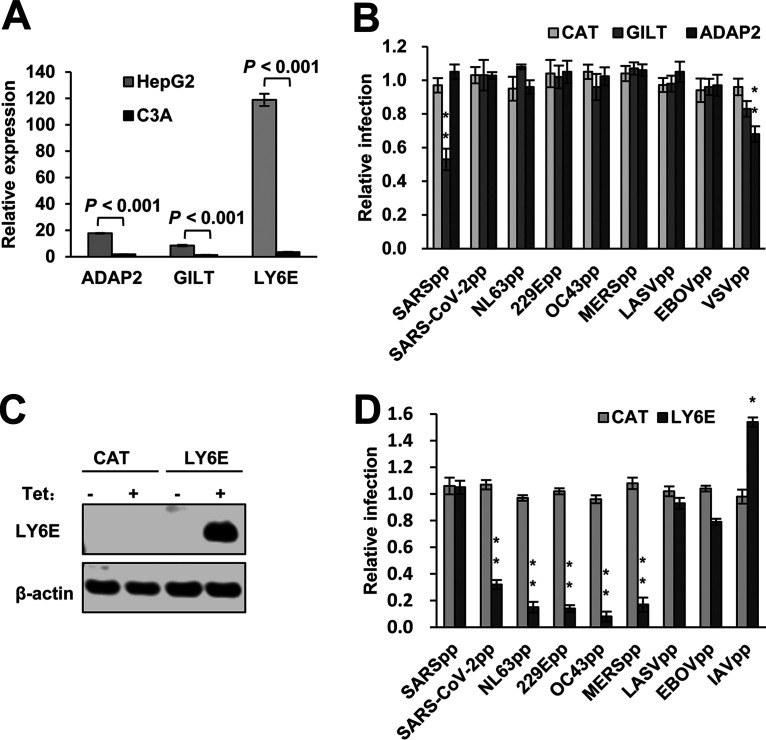
LY6E efficiently suppresses human coronavirus spike-protein-mediated entry. (A) Levels of Ly6E, GILT, and ADAP2 mRNA expression in HepG2 and C3A cells were determined by qRT-PCR assays and normalized to the level of GAPDH. (B) Flp-In T-Rex 293-derived cell lines expressing control protein CAT, GILT, or ADAP2 were cultured in the absence or presence of tet for 24 h. The cells were infected with HCoV-OC43pp and other indicated pseudoviral particles and intracellular luciferase activity were determined at 48 hpi. Relative infection is the ratio of luciferase activity in the same cells cultured in the presence of tet over that in the absence of tet. The error bars refer to standard deviations (*n* = 4). (C) Flp-In T-Rex 293-derived cell line expressing a control protein CAT or LY6E were cultured in the absence or presence of tet. Cells were harvested at 24 h after the addition of tet. The cellular expression of LY6E was detected by a Western blot assay. β-actin served as a loading control. (D) Flp-In T-Rex 293-derived cell lines expressing LY6E were cultured in the absence or presence of tet for 24 h. The cells were then infected with lentiviral particles pseudotyped with the envelope protein of the indicated viruses. Luciferase activities were determined at 48 hpi. Relative infection is the ratio of luciferase activity in the same cells cultured in the presence of tet over that in the absence of tet. The error bars refer to standard deviations (*n* = 4). **, *P* < 0.001 compared to the control cells expressing CAT.

**FIG 5:**
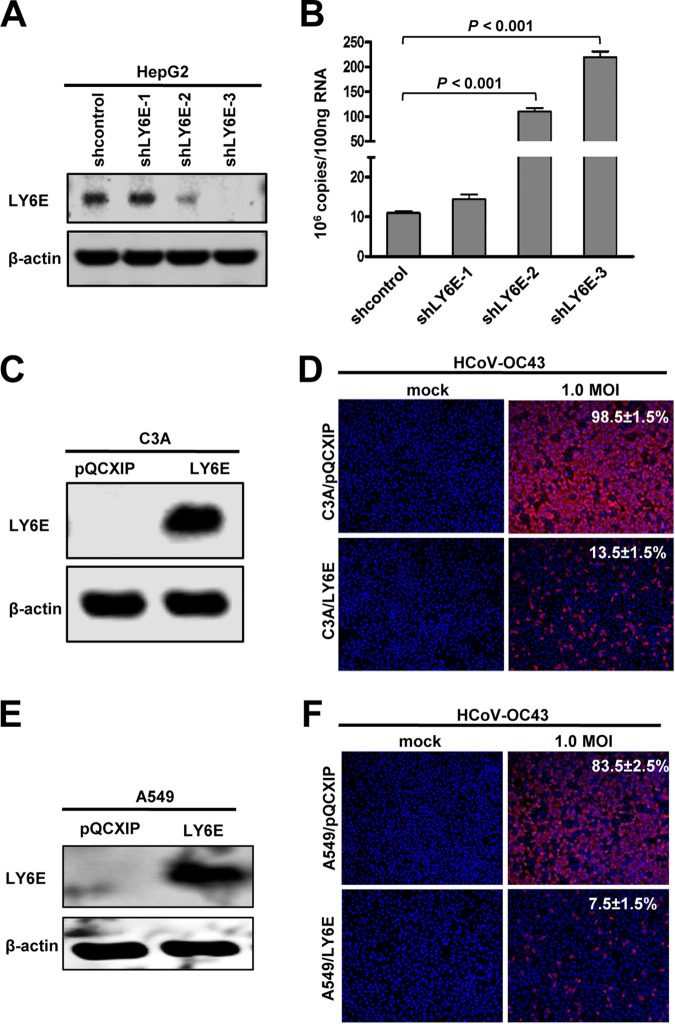
LY6E inhibits HCoV-OC43 infection in human hepatoma (HepG2 and C3A) and lung cancer (A549) cells. (A) HepG2 cells were stably transduced with scramble shRNA or shRNA targeting LY6E mRNA. The level of cellular LY6E expression was determined by Western blotting using a rabbit polyclonal antibody against LY6E. β-actin served as a loading control. (B) HepG2 cells stably expressing the scramble shRNA or LY6E-specific shRNA were infected with HCoV-OC43 at an MOI of 1.0. Cells were harvested at 24 hpi and intracellular viral RNA was quantified by qRT-PCR assay and presented as copies per 100 ng total RNA. Error bars indicate standard deviations (*n* = 4). Differences in viral RNA between scramble or LY6E-specific shRNA-expressing cells were analyzed statistically (**, *P* < 0.001; Student’s *t* test). (C to F) C3A or A549 cells were stably transduced with an empty retroviral vector (pQCXIP) or retroviral vector expressing LY6E and infected with HCoV-OC43 at the indicated MOI. The expression of LY6E in the cell lines was confirmed by a Western blot assay. β-actin served as a loading control (C and E). The cells were fixed at 24 hpi. The infected cells were visualized by IF staining of HCoV-OC43 N protein (red); cell nuclei were visualized by DAPI staining (D and F).

In order to identify host cellular proteins that may enhance HCoV-OC43 infection of C3A cells or suppress the virus entry into HepG2 cells, we first compared the expression of several cellular genes with known activity to restrict or enhance virus entry into target cells. As shown in Fig. 4A, we found that ADAP2, GILT, and LY6E mRNAs were expressed at significantly higher levels in HepG2 cells. While the expression of ADAP2 and GILT did not inhibit HCoV-OC43pp infection (Fig. 4B), expression of LY6E in Flp-In TREx 293 cells efficiently suppressed the infection of lentiviral particles pseudotyped with the envelope glycoproteins of all the human CoVs, except for SARSpp (Fig. 4C and D). In agreement with previous reports (38, 46), while LY6E enhanced the infection of IAVpp, expression of GILT inhibited SARSpp infection (Fig. 4D). To further confirm the role of LY6E in HCoV-OC43 infection, we showed that while reducing the expression of LY6E in HepG2 cells by shRNA knockdown significantly increased the viral RNA in HCoV-OC43-infected cells by 10- to 20-fold (Fig. 5A and B), ectopic expression of LY6E in C3A (Fig. 5C and D) or A549 (Fig. 5E and F) cells reduced their susceptibility to the virus infection by approximately 10-fold. The results presented above imply that LY6E is a restriction factor for human CoVs and responsible for the differential susceptibility of C3A and HepG2 cells to HCoV-OC43 infection.

### LY6E restriction of human coronavirus entry depends on GPI anchor and the evolutionally conserved L36 residue.

**FIG 6:**
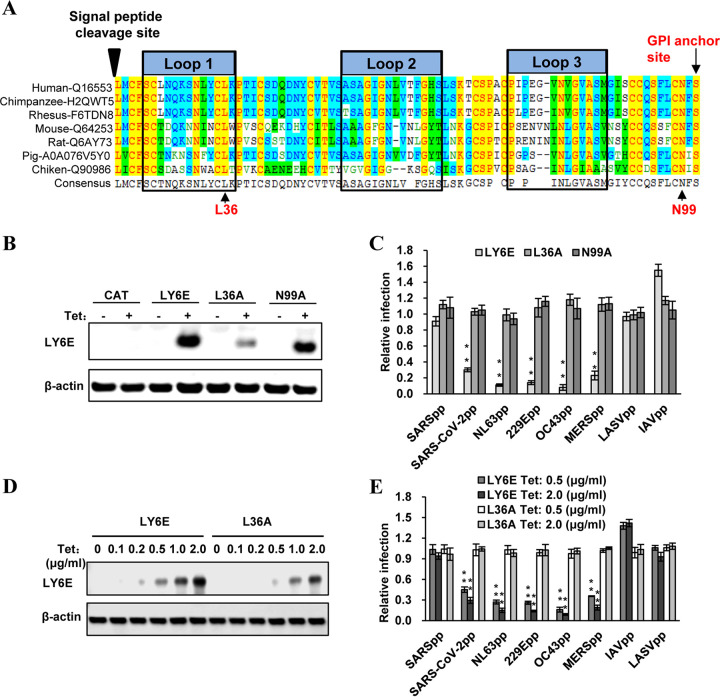
Identification of critical structural motifs essential for LY6E to restrict human coronavirus entry. (A) The amino acid sequence alignment of LY6E from multiple vertebrate species is presented, in which the “three finger-fold” structure is highlighted with black boxes. The conserved L36 as well as the GPI anchor and N99 glycosylation sites are indicated. (B) Flp-In T-Rex 293-derived cell lines expressing a control protein CAT or wild-type or mutant LY6E were cultured in the absence or presence of tet for 24 h. LY6E expression was detected by a Western blot assay in which β-actin served as a loading control. (C) Flp-In T-Rex 293-derived cell lines expressing the wild-type or mutant LY6E were cultured in the absence or presence of tet for 24 h. The cells were then infected with the indicated pseudotyped lentivirus. Luciferase activities were measured at 48 hpi. Relative infection is the ratio of luciferase activity in the same cells cultured in the presence of tet over that in the absence of tet. The error bars refer to standard deviations (*n* = 4); **, *P* < 0.001 compared to mutant LY6E. (D) Flp-In T-Rex 293-derived cell lines expressing the wild-type or mutant LY6E were cultured in medium with indicated concentrations of tet for 24 h. LY6E expression was detected by a Western blot assay in which β-actin served as a loading control. (E) Flp-In T-Rex 293-derived cell lines expressing the wild-type or mutant LY6E (L36A) were cultured with or without the indicated concentrations of tet for 24 h. The cells were then infected with the indicated pseudotyped lentivirus. Luciferase activities were measured at 48 hpi. Relative infection is the ratio of luciferase activity in the same cells cultured in the presence of tet over that in the absence of tet. The error bars refer to standard deviations (*n* = 4); **, *P < *0.001 compared to mutant LY6E.

LY6E is a member of the LY6/uPAR superfamily (47). Like most LY6 family members, LY6E contains 10 cysteines that form a highly conserved, three-finger folding motif through disulfide bonding and localizes on the plasma membrane of cells via glycosylphosphatidylinositol (GPI) anchoring. LY6E is ubiquitously expressed in many cell types and functions in modulation of cell signal transduction (41). Recent studies revealed that human LY6E promotes the entry of HIV (48, 49) and multiple enveloped RNA viruses from several viral families (46). Moreover, the enhancement of RNA viral infection is a conserved function of all the mammalian LY6E orthologs examined thus far. Particularly, substitution of the evolutionally conserved residue L36 with alanine (A) completely abolished the viral enhancement activity of LY6E (46) (Fig. 6A). Interestingly, we found that the L36A substitution also abolished the ability of LY6E to restrict the entry of human CoVs (Fig. 6B and C). However, the poor expression of this mutant protein may contribute to the failure of inhibiting virus entry. To ascertain the functionality of L36A mutant protein, Flp-In T REx 293-derived cell lines expressing wild-type or mutant LY6E were cultured in a serial concentration of tetracycline (tet) to induce different levels of wild-type and mutant LY6E expression. As shown in Fig. 6D and E, while the level of wild-type LY6E in cells treated with 0.5 μg/ml of tet was slightly lower than the level of L36A LY6E in cells treated with 2 μg/ml of tet, the wild-type LY6E efficiently inhibited the infection of all the tested HCoVpp, except for SARSpp, but the mutant LY6E failed to inhibit any of the tested HCoVpp under these conditions. These results thus confirmed that the L36 residue is indeed essential for LY6E modulation of HCoV entry. As anticipated, the N99A substitution that disrupts the addition of GPI anchor also abrogated the inhibitory effects of LY6E on human CoV entry (Fig. 6B). These results indicate that proper interaction of LY6E with other viral/cellular components via the conserved residue L36 and localization in the specific cell membrane microdomians are required for LY6E restriction of human CoV entry.

### Activation of CoV entry by TMPRSS2 expression fails to evade LY6E restriction of CoV entry.

**FIG 7:**
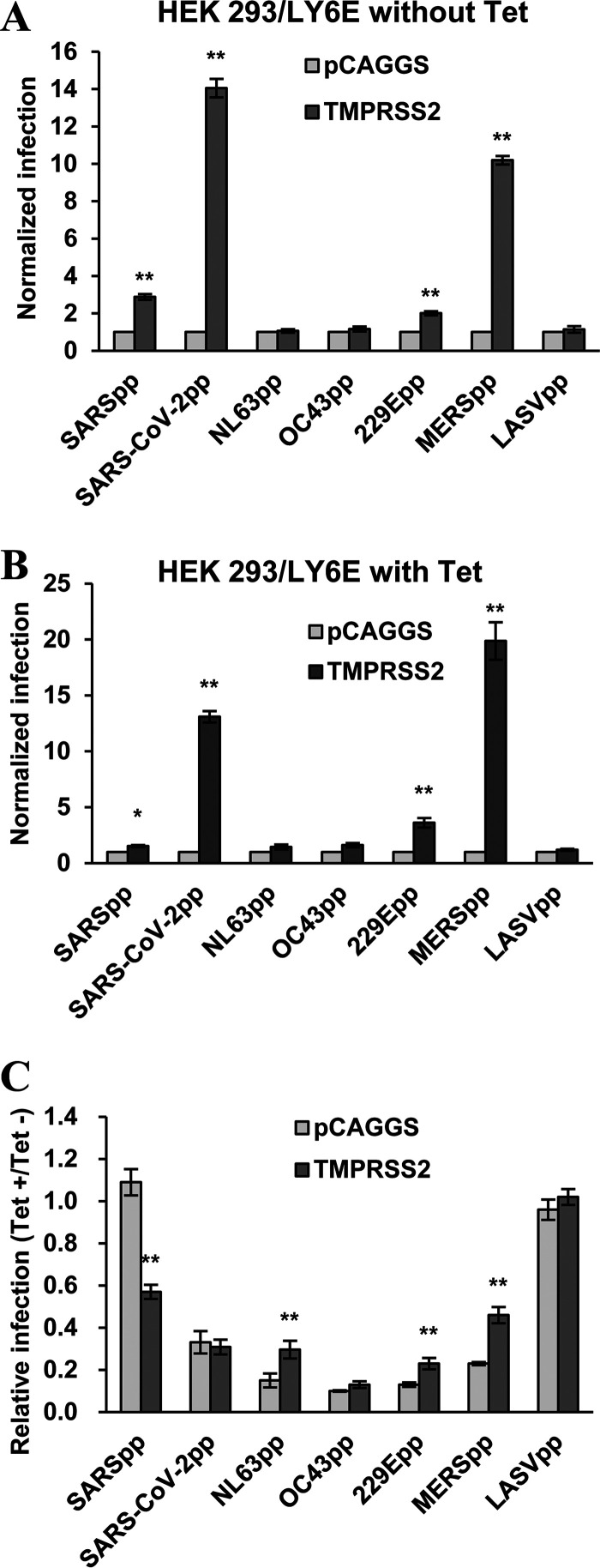
LY6E inhibits TMPRSS2-enhanced entry of human coronaviruses. Flp-In T-Rex 293-derived cell lines expressing LY6E were transfected with a control vector (pCAGGS) or a plasmid expressing human TMPRSS2 and cultured in the absence or presence of tet for 24 h. The cells were then infected with the indicated pseudotyped lentivirus. Luciferase activities were measured at 48 hpi. (A) The effect of TMPRSS2 expression on pseudotyped virus infection is normalized to infection efficiency of the cells transfected with control vector plasmid (set as 1) in the cells cultured in the absence of tet. (B) The effect of TMPRSS2 expression on pseudotyped virus infection is normalized to infection efficiency of the cells transfected with control vector plasmid (set as 1) in the cells cultured in the presence of tet. (C) Relative infection refers to the ratio of the luciferase activity in the cells cultured in the presence of tet over that in the cells cultured in the absence of tet. Error bars indicate the standard deviation (*n* = 4); **, *P* < 0.001 compared to cells transfected with the pCAGGS vector.

It was reported by us and others that expression of the cell membrane-associated serine protease TMPRSS2 enhances SARS-CoV and SARS-like bat CoV entry (32–34). More importantly, the TMPRSS2-enhanced entry can evade IFITM3 restriction (50), presumably because the cellular protease activates the viral fusion at the cell surface or early endosomes where IFITM3 expression is at a relatively lower level and thus fails to inhibit viral fusion. To determine the effects of TMPRSS2 expression on LY6E restriction of human CoV entry, the Flp-In TREx 293-derived cell line expressing LY6E was transfected with a control vector (pCAGGS) or a plasmid expressing human TMPRSS2 and cultured in the absence or presence of tet for 24 h. The cells were then infected with the indicated pseudotyped lentiviruses. As shown in Fig. 7A, in the absence of tet to induce LY6E, expression of TMPRSS2 significantly enhanced the infection of SARSpp, SARS-CoV-2pp, MERSpp, and 229Epp, but not the infection of other human CoVpp and LASVpp. However, in the presence of tet to induce LY6E expression, the TMPRSS2-enhanced infection of SARSpp, but not SARS-CoV-2pp, MERSpp, and 229Epp, was significantly diminished (Fig. 7B). As summarized in Fig. 7C, expression of LY6E in the absence or presence of TMPRSS2 significantly inhibited the infection of all the COVpp, except for SARSpp, which was only significantly inhibited in the presence of TMPRSS2 expression. Therefore, unlike IFITM3, expression of TMPRSS2, a cellular protease that is known to activate the fusion activity of SARS-CoV spike proteins at the cell surface or early endosomes (33, 51), does not prevent, but rather confers susceptibility to, LY6E restriction of SARS-CoV entry.

### Amphotericin B treatment does not compromise LY6E restriction of human CoV entry.

**FIG 8:**
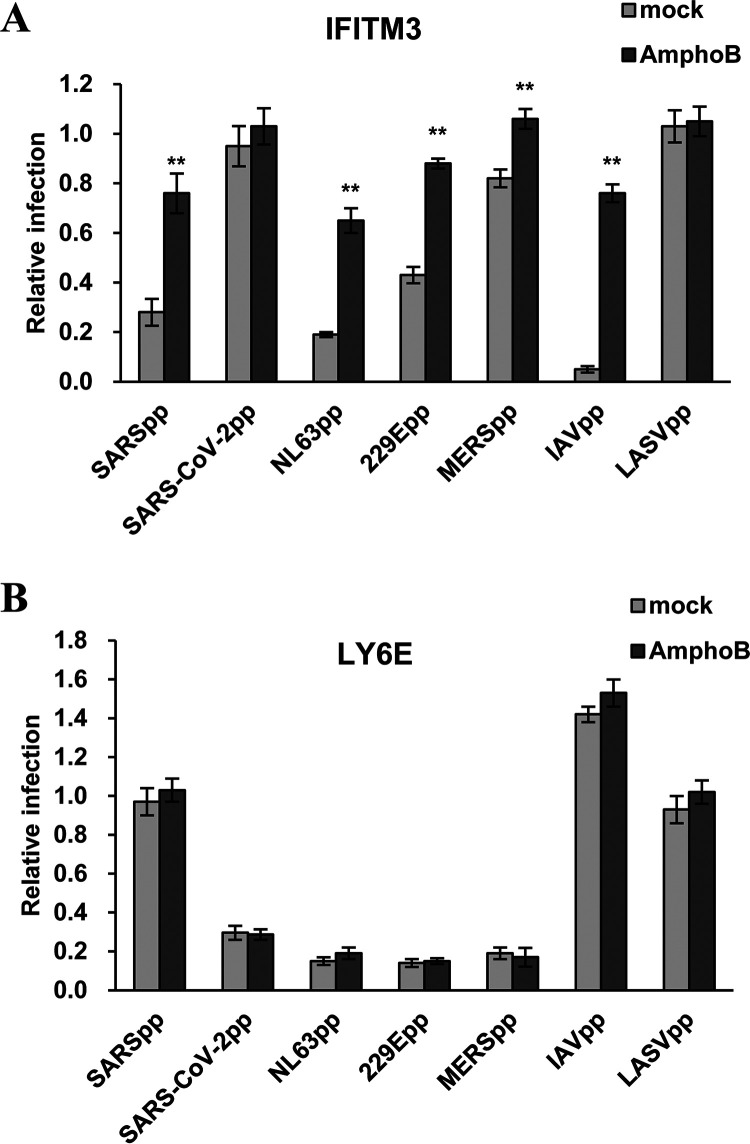
Amphotericin B treatment compromises IFITM3 inhibition of human coronavirus entry, but have no impact on Ly6E inhibition of human coronavirus entry. Flp-In T-Rex 293-derived cell lines expressing IFITM3 (A) or LY6E (B) were cultured in the absence or presence of tet for 24 h. The cells were then infected with the indicated pseudotyped lentivirus in the presence or absence of 1 μM AmphoB. Luciferase activity was measured at 48 h postinfection. Relative infection is the ratio of luciferase activity in the same cells cultured in the presence of tet over that in the absence of tet. The error bars refer to standard deviations (*n* = 4); **, *P* < 0.001 compared to mock treatment.

Amphotericin B (AmphoB) is an antifungal medicine that binds with ergosterol in fungal cell membranes, forming pores that cause rapid leakage of monovalent ions and subsequent fungal cell death. AmphoB can also bind to cholesterol in mammalian cell membranes, albeit at a lesser affinity than to fungal ergosterol (52). The cholesterol-rich plasma membrane microdomains known as lipid rafts play important roles in the entry and egress of many enveloped viruses (53, 54). Particularly, AmphoB treatment has been shown to significantly compromise IFITM restriction of IAV entry (55) and attenuate IFITM enhancement of HCoV-OC43 infection (42). In this study, we further demonstrated that AmphoB treatment also efficiently attenuated the restriction of IFITM3 on the infection of SARSpp, MERSpp, NL63pp, 229Epp, and IAVpp, but not LASVpp (Fig. 8A). However, AmphoB treatment altered neither the restriction activity of LY6E on the infection of human CoV spike protein-pseudotyped lentiviruses nor the enhancement of LY6E on IAVpp infection (Fig. 8B). These results strongly imply that LY6E modulates virus entry is most likely not through modulation of cholesterol function in membrane fusion.

## DISCUSSION

LY6E was initially identified as a cell surface marker to discriminate immature from mature thymocyte subsets (56). The primary function of LY6E has been associated with immune regulation, specifically in modulating T cell activation, proliferation, and development (57). In addition to lymphocytes, LY6E mRNA can also be detected in liver, spleen, uterus, ovary, lung, and brain. The expression of LY6E can be induced by type I IFN in a cell-type specific manner (56). However, LY6E is not a typical antiviral effector protein. Instead, LY6E was reported to promote the infection of enveloped RNA viruses from several viral families (46) and modulates HIV-1 infection in a manner dependent on the level of CD4 expression in target cells (48, 49). Our finding that LY6E restricts human CoV infection and our characterization of its antiviral effects shed new light on the mode of LY6E action on virus entry in general.

First, either the enhancement or restriction of virus entry by LY6E depends on its GPI anchor (Fig. 6) (46, 48, 49). GPI-anchored proteins are preferentially located in lipid rafts, the plasma membrane microdomains enriched in glycosphingolipids and cholesterol, as well as protein receptors or ligands. Lipid rafts are considered to compartmentalize membrane processes by facilitating the interaction of protein receptors and their ligands/effectors to modulate membrane functions, such as signal transduction, membrane fusion, vesicle budding, and trafficking. Lipid rafts are also involved in the entry and egress of many viruses. For instance, both HIV-1 receptors (CD4) and coreceptors are localized in lipid rafts. Yu and colleagues elegantly demonstrated recently that LY6E enhances HIV-1 infection of CD4^+^ T cells and monocytic THP1 cells by promoting the expansion of viral fusion pores induced by HIV-1 Env (49). Furthermore, LY6E was found to be the receptor of mouse endogenous retroviral envelope Syncytin-A, and interaction of LY6E with Syncytin-A induces syncytiotrophoblast fusion and placental morphogenesis (58, 59). However, Mar and colleagues showed that LY6E enhances IAV infection of cells by promoting a viral replication step after viral nucleocapsid escape from endosomes, but before viral RNP nuclear translocation, i.e., most likely the uncoating of nucleocapsids (46). Interestingly, the results presented in Fig. 4 indicate that LY6E significantly enhanced the infection of IAVpp, suggesting that the LY6E enhancement of IAV infection is, at least in part, through promoting the entry into target cells, possibly also by enhancing viral fusion. Considering the broad inhibitory effects of LY6E on human CoVs and its fusogenic or fusion-modulating activity, we speculate that LY6E might inhibit the membrane fusion triggered by CoV spike proteins. However, the role of LY6E on endocytosis and endocytic vesicle trafficking cannot be ruled out. These hypotheses are under investigation.

Second, in addition to the GPI anchor, the evolutionally conserved amino acid residue L36 is also required for both the enhancement and restriction of virus entry into target cells by LY6E (Fig. 6) (46). It can be speculated that this specific residue may mediate an interaction with other cellular membrane proteins to module viral entry. The fact that LY6E enhances viral infectivity in a cell type-specific manner, with the strongest phenotype in cells of fibroblast and monocytic lineages (46), does indicate the involvement of other host cellular factors. Variations in the abundance of expression, as well as the localization of LY6E and its associated proteins or lipids, may explain the differential effects of LY6E on the infection of different viruses in different cell types (Fig. 4 and 7). However, LY6E enhancement of RNA virus infection appears to be independent of the type I interferon response and other ISG expression (46). Particularly, enhancement of viral infection in Huh7.5 cells that do not have basal levels of IFITM protein expression indicates that LY6E enhancement of RNA viral infection is most likely not through modulating the function of IFITM proteins (42). This notion is further supported by the finding that LY6E- and Syncitin-A-mediated syncytiotrophoblast fusion can be inhibited by IFITM proteins (60, 61).

Third, studying the effects of LY6E on HIV-1 infection of CD4 low-expressing cells, such as Jurkat T cells and primary monocyte-derived macrophages, revealed that HIV-1 entry was inhibited by LY6E (48). This appears due to the LY6E-induced reduction of lipid raft-associated CD4 on the surface of these cells. It was demonstrated that LY6E can promote CD4 endocytosis and mobilize lipid raft-associated CD4 molecules to non-raft microdomains. Such a receptor downregulation significantly reduced HIV-1 binding and infection of CD4 low-expressing cells (macrophages), but did not significantly impact the binding of HIV-1 to CD4 high-expressing cells, which allows for LY6E to predominantly enhance HIV-1 infection of CD4^+^ T lymphocytes by promotion of membrane fusion (48). It is, therefore, possible that LY6E inhibition of human CoV infection is due to the downregulation of lipid raft-associated CoV receptors. However, the differential effects of LY6E on the infection of SARSpp, SARS-CoV-2pp, and NL63pp, which all share the ACE2 receptor, do not support such a hypothesis (Fig. 4).

Fourth, the differential effects of LY6E on the entry mediated by the spike proteins of two closely related human coronaviruses, SARS-CoV and SARS-CoV-2, in HEK 293 cells are very striking. The spike proteins of these two viruses share 76% amino acid sequence identity (8, 10) and use the same cellular receptor (ACE2) to drive virus entry process. Particularly, two consecutive proteolytic cleavages at the boundary of the S1/S2 domains and immediately upstream of the fusion peptide in the S2 domain (S2’) are essential to trigger the fusion of viral envelope with target cell plasma membrane (early entry) and/or endo-lysosomal membranes (late entry) (62, 63). Distinct from SARS-CoV spike protein, SARS-CoV-2 spike protein has a unique 4-amino-acid insertion at the S1/S2 boundary, which generates a polybasic furin cleavage site (16, 64). Recent studies indicated that the first proteolytic activation step of SARS-CoV-2 spike protein is catalyzed by furin in virally infected cells during virion secretion (16, 65). The activation of membrane fusion by cleavage at the S2’ site can be catalyzed by many cell membrane-associated proteases for early entry or, alternatively, by endosomal cathepsins for late entry (16, 65). Due to the lack of a furin cleavage site for preactivation during virion secretion and/or lower expression of plasma membrane proteases at the target cells, SARS-CoV spike protein usually mediates late entry (38, 66, 67). However, under the conditions of exogenous trypsin treatment or overexpression of TMPRSS2 or other proteases, SARS-CoV spike protein can also be activated at the plasma membrane to mediate early entry (66). Based on the differential sensitivity of viruses to LY6E and IFITM3 and the subcellular localization of these two restriction factors, we favor the hypothesis that the plasma membrane LY6E primarily restricts the early entry, but the endosomal IFITM3 mainly restricts the late entry of coronaviruses. This hypothesis is supported by our observation that overexpression of plasma membrane-associated serine protease TMPRSS2 in HEK293 cells conferred the sensitivity of SARSpp infection to LY6E, but evaded the restriction of IFITMs (Fig. 7) (50).

Finally, the finding that LY6E inhibition of human CoV entry cannot be compromised by AmphoB treatment strongly indicates that LY6E modulates virus entry via a distinct mechanism from that used by IFITM proteins (Fig. 8). AmphoB can bind cholesterol in cell membranes to increase membrane fluidity and planarity and, consequently, rescue IFITM inhibition of virus entry (55). Interestingly, AmphoB can neutralize the antiviral effects of IFITM2 and IFITM3, but has little effect on IFITM1 restriction of virus entry (55). While IFITM1 is predominantly located in the plasma membrane or early endosomes, IFITM2 and 3 are mainly localized in the later endosomes and lysosomes. Due to their differential subcellular localization, IFITM1 mainly restricts the viruses that enter the cells at the cell surface or in the early endosomes, such as parainfluenza viruses and hepatitis C virus (68, 69), whereas IFITM2 and 3 primarily restrict the infection of viruses that enter the cells at later endosomes and/or lysosomes (43, 70, 71). Because AmphoB is endocytosed quite rapidly, leading to its concentration in the late endosomes and lysosomes, it more efficiently alleviates the effect of IFITM2 and 3, but not IFITM1, on virus entry (55). Similarly, the failure of AmphoB to attenuate the antiviral effects of LY6E against human CoVs is most likely due to the predominant cell surface localization of LY6E and inhibition of an early entry of CoVs.

In summary, while it is very interesting to know that LY6E is capable of modulating the entry of many RNA viruses, we have only begun to uncover the mechanisms of this fascinating host factor and define its pathobiological role in virus infection (41, 47). Further understanding of the role and mechanism of LY6E in viral infections will establish a scientific basis for development of therapeutics to harness its function for the treatment of viral diseases.

## MATERIALS AND METHODS

### Cell cultures.

Human hepatoma cell lines HepG2 and C3A, a subclone of HepG2 (ATCC HB-8065), were purchased from the ATCC and cultured in Dulbecco’s modified Eagle medium (DMEM)/F12 medium supplemented with 10% heat-inactivated fetal bovine serum (FBS) (Invitrogen). Lung cancer cell line A549 was obtained from the ATCC and maintained in DMEM supplemented with 10% FBS. GP2-293 and Lenti-X 293T cell lines were purchased from Clontech and cultured in DMEM supplemented with 10% FBS and 1 mM sodium pyruvate (Invitrogen). Flp-In TREx 293 cells were purchased from Invitrogen and maintained in DMEM supplemented with 10% FBS, 10 μg/ml blasticidin (Invitrogen), and 100 μg/ml zeocin (Invivogen) (72). Flp-In TREx 293-derived cell lines expressing LY6E, GILT, ADAP2, or IFITM3 were cultured in DMEM supplemented with 10% FBS, 5 μg/ml blasticidin, and 250 μg/ml hygromycin.

### Viruses.

HCoV-OC43 (strain VR1558) was purchased from the ATCC and amplified in HCT-8 cells according to the instructions from the ATCC. Virus titers were determined by a plaque assay as described previously (42). Briefly, HCT-8 cells were seeded into 24-well plates at a concentration of 1 × 10^5^ cells per well. Twenty-four hours later, confluent HCT-8 cells were infected with 200 μl of OPTI-MEM containing a serial 10-fold dilution of viral stock for 2 h at 33°C. After removal of the inoculum, HCT-8 cells were washed with RPMI 1640 medium and overlaid with RPMI 1640 medium containing 0.5% methylcellulose and cultured at 33°C for 4 to 5 days. Plaques were monitored and counted under the microscope.

### Antibodies.

Monoclonal antibody against the FLAG tag (anti-FLAG M2) and β-actin were purchased from Sigma (catalog numbers F1804 and A2228, respectively). Monoclonal antibody against human IFITM1 (catalog number 60047-1), rabbit polyclonal antibody against human IFITM3 (catalog number 11714-1-AP), which also efficiently recognizes IFITM2 and weakly cross-reacts with IFITM1, were purchased from Proteintech Group, Inc. Mouse monoclonal antibody against HCoV-OC43 nucleocapsid (NP) protein was purchased from Millipore (catalog number MAB9012). Rabbit polyclonal antibody against human LY6E was obtained from Proteintech (catalog number 22144-1-AP).

### Plasmid construction.

The cDNA molecules of ADAP2 and LY6E were purchased from OriGene (catalog numbers RC207501 and RC211373, respectively) and cloned into the pcDNA5/FRT-derived vector as described previously (42). Ly6E and N-terminally FLAG-tagged human IFITM1, IFITM3, and their mutants were cloned into pQCXIP vector (Clontech) between the NotI and BamHI sites as previously described (42, 43). pcDNA5/FRT-derived plasmids expressing chloramphenicol acetyltransferase (CAT) and N-terminally FLAG-tagged human IFITM3 were reported previously (72–74).

Plasmids expressing HCoV-OC43 spike (S) and HE proteins, VSV G protein, H1N1 IAV (A/WSN/33) hemagglutinin (HA) and neuraminidase (NA), LASV GP protein, murine leukemia virus (MLV) envelope protein, HCoV-NL63, HCoV-229E, SARS-CoV, and MERS-CoV spike protein were described previously (75, 76). The codon-optimized (for human cells) SARS-CoV-2 spike gene, which is based on NCBI reference sequence YP_009724390.1, was purchased from GeneScript and cloned into the pCAGGS vector as described previously (77). pRS-derived retroviral vectors expressing a scramble shRNA and shRNA targeting the mRNA of human LY6E were obtained from OriGene (catalog number TR311641).

Plasmid pNL4-3.Luc.R-E- was obtained through the NIH AIDS Research and Reference Reagent Program (78, 79). Angiotensin I converting enzyme 2 (ACE2), aminopeptidase N (APN), and dipeptidyl peptidase-4 (DPP4) cDNA clones were obtained from Origene, and cloned into a pcDNA3 vector (Invitrogen) to yield plasmids pcDNA3/ACE2, pcDNA3/APN, and pcDNA3/DDP4, respectively (77).

### Packaging of pseudotyped retroviral particles.

The various viral envelope protein pseudotyped lentiviruses bearing luciferase reporter genes, the VSV G protein pseudotyped Moloney murine leukemia virus (MMLV)-derived retroviral vectors (pQCXIP) expressing wild-type and mutant human IFITM and/or LY6E, or the pRS vector-derived plasmids expressing a scrambled shRNA or shRNA specifically targeting human LY6E were packaged as reported previously (77). To package pseudotyped lentiviral particles, 2 × 10^6^ Lenti-X 293T cells were seeded into a 100-mm-diameter dish 1 day prior to transfection. Cells were cotransfected with 20 μg of pNL4.3.Luc.R-E- and 10 μg of plasmid expressing a virus envelope protein by using a calcium phosphate precipitation procedure. At 24 h posttransfection, the cells were replenished with 15 ml of complete DMEM. The culture supernatants were harvested at 48 h after transfection, filtered through a 0.45-μm pore sized PES syringe filter (Millipore), titrated with Lenti-X p24 rapid titer assay (TaKaRa Bio, catalog number 632200), and stored at −80°C until use. Each pseudotype was also titrated by infection of HEK293 cells transfected with the respective CoV receptor with a serial dilution of pseudotype preparations. The modulation of IFITM or LY6E on the transduction of a given pseudotype (20 ng p24-normalized pseudotype virus) was determined with a titrated amount of pseudotypes that yield luciferase signal between 10,000 to 1,000,000 light units per well of 96-well plates (77, 80). For a given pseudotype, the input of pseudoviral particles was consistent across all the experiments.

### Establishment of cell lines stably expressing wild-type and mutant IFITM or LY6E proteins or shRNA.

HepG2, C3A, or A549 cells in each well of 6-well plates were incubated with 2 ml of Opti-MEM medium containing pseudotyped retroviruses and centrifuged at 20°C for 30 min at 4,000 × *g*. Forty-eight hours postransduction, cells were cultured with medium containing 2 μg/ml of puromycin for 2 weeks. The antibiotic-resistant cells were pooled and expanded into cell lines stably expressing wild-type or mutant IFITM or LY6E proteins or shRNA targeting LY6E. Flp-In TREx 293-derived cell lines expressing IFITM, GILT, ADAP2, or LY6E proteins in a tetracycline (tet)-inducible manner were established as previously described (72, 74).

### Immunofluorescence.

To visualize HCoV-OC43-infected cells, the infected cultures were fixed with 2% paraformaldehyde for 10 min. After permeabilization with 0.1% Triton X-100, the cells were stained with a monoclonal antibody (541-8F) recognizing HCoV-OC43 NP protein. The bound antibodies were visualized by using Alexa Fluor 488-labeled (green) goat anti-mouse IgG or Alexa Fluor 555-labeled (red) goat anti-mouse IgG. Cell nuclei were counterstained with DAPI (4′,6-diamidino-2-phenylindole).

### Western blot assays.

Cells were lysed with 1× Laemmli buffer. An aliquot of cell lysate was separated on NuPAGE Novex 4% to 12% Bis-Tris Gel (Invitrogen) and electrophoretically transferred onto a nitrocellulose membrane (Invitrogen). The membranes were blocked with PBS containing 5% nonfat dry milk and probed with the desired antibody. The bound antibodies were visualized with IRDye secondary antibodies and imaging with LI-COR Odyssey system (LI-COR Biotechnology).

### Real-time RT-PCR.

HCoV-OC43 RNA was quantified by a quantitative reverse transcriptase PCR (qRT-PCR) assay as described previously (42). To determine the level of ISG mRNA, total cellular RNA was extracted using TRIzol reagent (Invitrogen) and the same amount of total cellular RNA was reverse-transcribed with SuperScript III kit ((Invitrogen). Quantitative RT-PCR was performed using iTaq universal SYBR green Supermix (Bio-Rad) with the following primers: LY6E, 5′-GTACTGCCTGAAGCCGACCATC-3′ and 5′-AGATTCCCAATGCCGGCACTAG-3′; ADAP2, 5′-AGCTGTCATCAGCATTAAG-3′ and 5′-ACTATCTCCTTCCCACTTTC-3′; GILT, 5′-AATGTGACCCTCTACTATGAAG-3′ and 5′-ACGCTGGTGCCCTACGGAAACG-3′; GAPDH, 5′-GAAGGTGAAGGTCGGAGTCAAC-3′ and 5′-CAGAGTTAAAAGCAGCCCTGGT-3′. Gene expression was calculated using the threshold cycle (2^-△△CT^) method, normalized to GAPDH as described previously (31, 40).

### Luciferase assays.

Flp-In TREx 293-derived IFITM-expressing cell lines were seeded into 96-well plates with black walls and clear bottoms and transfected with an empty vector plasmid or plasmids encoding ACE2, APN, or DPP4 to express viral receptors. For Huh7.5-derived IFITM-expressing cell lines, cells were seeded into black-walled 96-well plates. Cells were infected at 24 h posttransfection or infected with desired pseudotyped lentiviral particles for 2 h, and then replenished with fresh medium. Two days postinfection, the media were removed and cells were lysed with 20 μl/well of cell lysis buffer (Promega) for 15 min, followed by adding 50 μl/well of luciferase substrate (Promega). The firefly luciferase activities were measured by luminometry in a TopCounter (Perkin Elmer) (77).

### Statistical analyses.

All the experiments were repeated at least three times. Differences between control samples and tests were statistically analyzed using Student’s *t* tests or one-way analysis of variance (ANOVA). *P* values of less than 0.05 were considered statistically significant.
